# Perception-reality bias: the differences in government trust across income groups

**DOI:** 10.3389/fpsyg.2023.1157828

**Published:** 2023-07-20

**Authors:** Kezhong Jiang, Yiqiang Zhang, Victor Shi

**Affiliations:** ^1^School of Public Finance and Administration, Shanghai Lixin University of Accounting and Finance, Shanghai, China; ^2^Lazaridis School of Business and Economics, Wilfrid Laurier University, Waterloo, ON, Canada

**Keywords:** subjective evaluation, objective criteria, bias, middle-income group, government trust

## Abstract

This paper first measures and compares the size of middle-income groups in China based on the subjective income evaluation method and the objective criteria. Second, it empirically investigates the differences in government trust of different income groups defined by the subjective evaluation method and the objective criteria. It is found that there is a significant difference between the results of the subjective evaluation of income and objective criteria. Compared with individuals in the middle-income group, individuals in the low-income group have a significantly worse overall evaluation of local government and a considerably lower trust in local government officials. On the other hand, individuals in the high-income group have a substantially better assessment of local government and a significantly higher trust in local government officials. However, the differences in trust in government across income groups defined by objective criteria are insignificant overall. In terms of policy insights, the effect of targeting low-income groups determined by subjective evaluation may be more effective in improving people’s trust in the government.

## 1. Introduction

The middle-income group is a constant research topic in the political economy of the developed countries represented by the United States, especially after the economic crisis. In the 1970s and 1980s, the economy of developed countries fell into “stagflation.” Thurow (1984) in the New York Times aroused widespread concern and discussion about the fate of middle-income groups in the context of high unemployment and inflation. During 2007–2009, with the outbreak of the world economic crisis, then U.S. President Barack Obama set up the White House Middle Class Task Force under the responsibility of Vice President Joe Biden to deal with the impact of the economic crisis on the middle-income group. Obama emphasized that “… the strength of our economy can be measured by the strength of our middle class, and a strong middle class equals a strong America, one cannot be without the other …” (The White House Task Force on the Middle Class, 2010). Furthermore, developing countries have experienced decades of relatively peaceful and high economic growth and the rise of middle-income groups, and their importance to economic and social development has received increasing attention, especially in this century, and studies related to middle-income groups in developing countries have gradually increased (Rasch, 2017; Manzano et al., 2020).

Traditionally, most studies have used objective criteria to define middle-income groups, but there is no consensus on whether absolute, relative, or mixed criteria should be chosen and what interval should be selected for a particular criterion. Based on the reality of China, limited and inaccurate income statistics remain challenges in the selection of objective criteria. In rural and some underdeveloped areas of China, cash and in-kind economies are prevalent, informal employment is common, income information systems are missing, and household surveys are costly and low-quality (Zhang, 2017). Chinese residents, especially those in high-income groups, tend to underreport or conceal their income. The income levels of Chinese residents vary significantly between urban and rural areas and between regions, making it difficult to determine an objective standard that applies to both urban and rural areas (Li and Xu, 2017). Different objective criteria for defining middle-income groups may lead to different measurement results (Rasch, 2017; Weng and Wang, 2019). And unclear objectives may lead to the problem that policies to expand middle-income groups cannot be implemented.

Unlike objective criteria, subjective evaluations of socioeconomic status do not rely on specifically defined intervals, do not require income surveys, are simple to operate, and can be experimentally manipulated.^1^ The method has recently emerged in the study of social groups (Rubin et al., 2014; Yang et al., 2022). Some studies have shown that it is not the objective socioeconomic status, but the subjective evaluation of an individual’s socioeconomic status that has a substantial effect on their well-being and health status (Cohen et al., 2008; Tan et al., 2020). The main reason for this is that a key factor in why an individual’s economic and social status affects behavior, cognition, etc., is the perception of their relative socioeconomic status (Dietze and Knowles, 2016; Kraus et al., 2017; Lee et al., 2018). When the results (reality) defined by objective criteria are not consistent with the subjective evaluation (perception) of individuals’ socioeconomic status, the “label” of the group defined by objective criteria may lose its meaning. In other words, the effectiveness of policies targeting different income groups defined by objective criteria may be significantly reduced.

In this paper, first, based on the subjective income evaluation method and the objective criteria method, the size of middle-income groups in China is measured and compared. Second, we follow the classical hypothesis and research on social class theory on the existence of discontinuous social “class fragments” and the differences in lifestyles, social cognition, political attitudes and political participation of members of different social classes in Bourdieu (1984, 1985, 1987). We conduct an empirical study on the differences in trust in government among different income groups in China. Our research results show that although it is impossible to judge whether the subjective evaluation method is better or worse than the objective criterion method, the subjective evaluation method can be considered as a supplement or alternative to the criterion for defining middle-income groups in China when the objective criterion is difficult or controversial. A major policy insight of this paper is that it may be more effective to target different income groups defined by subjective evaluation methods to improve people’s trust in the government.

## 2. Literature review

Traditionally, most studies have used objective criteria to define middle-income groups, but there is no consensus on whether absolute, relative, or mixed criteria should be chosen, and what measurement interval should be chosen under a particular criterion. Among them, developed countries more often choose relative or mixed criteria to define middle-income groups. For example, developed countries widely use the 75–125% median income range and 75–150% range as the criteria for determining the middle-income group (Pressman, 2007; Derndorfer and Kranzinger, 2021). Some studies combine observable multidimensional indicators such as occupation, income, assets, and education to define middle-income groups (Gilbert, 2014; Fan and Zan, 2020). Developing countries more often choose absolute or mixed criteria to define middle-income groups (Birdsall, 2010; Ravallion, 2010). The use of relative criteria for defining middle-income groups is more controversial in developing countries. For example, Vanneman and Dubey (2013) found that in India, if the 75–200% median income range is used as the criterion for defining the middle-income group, the measure includes many low-income groups earning less than $2 per day.

The absolute criteria mainly reflect the size of the population in a specific income range and its proportion, which is more suitable for developing or low- and middle-income countries. They can be used to explore the benefits of economic development for different income groups in these countries. It is also suitable for international comparative analysis, comparing the speed of economic development of different countries and the benefits of different economic development models. The relative criterion mainly reflects the size of the population with income in a specific quantile range and its proportion, which is more suitable for developed countries, where the level of economic development is higher and the size of middle-income groups accounts for a larger proportion of the population. Moreover, the relative criterion can explore the changes in the size of different groups brought about by fluctuations in income distribution in these countries.

The criteria for defining China’s middle-income group are based on the fact that China has entered the ranks of middle-income countries with the dual characteristics of a growing and improving society. Hence, both the goal of moving toward a high-income country and the goal of achieving common prosperity should be considered. It is relevant to measure the size of China’s middle-income group, both in absolute and relative terms (Li, 2017). In the literature, several studies adopt the absolute criteria (Li and Xu, 2017; Liu and Xu, 2017; Wang, 2020) and several adopt the relative criteria (Long, 2012, 2015; Wu and Chang, 2018; Ruan et al., 2021). Even though specific criteria are used, the specific measurement interval varies greatly. Taking the absolute criteria as an example, Li and Xu (2017) used *per capita* household disposable income of 20,000–67,000 yuan, *per capita* annual household income of 35,000–120,000 yuan, and annual household income of 69,000–236,000 yuan, respectively, as the criteria for defining middle-income groups. In terms of the relative criteria, Wu and Chang (2018) used the median income 75–125% range, and Long (2012, 2015) used the median income 75–125% and 50–150% range as the definition criteria of the middle-income group.

The measurement results of the size of middle-income groups vary greatly depending on the choice of the objective criteria (Rasch, 2017; Weng and Wang, 2019). And the lack of clear objectives may lead to the dilemma that policies to expand middle-income groups cannot be implemented. This has led researchers to reflect on the shortcomings of objective criteria. First, objective criteria need to be constructed accordingly based on the totality of the measured objects, and social groups are conceptualized as absolute or relative indicators, so how many categories should be included, and how the upper and lower limits of different classes should be chosen are highly controversial issues (Bourdieu, 1987; Li and Xu, 2017). Objective criteria are also limited to a specific time and context, and it is difficult to generalize them outside their particular time and context, which is the key reason why it is difficult to construct an international standard for defining middle-income groups. Second, an important indicator of objective criteria to define middle-income groups is the income level of individuals or their households. Still, in many cases, income statistics are complicated and inaccurate. For example, Jetten et al. (2008) studied the adjustment to university life of children from different income groups in European countries. They found that 51% of respondents were unable to complete a measure of household income. In developing countries, including rural and some underdeveloped areas of China, cash and in-kind economies prevail, informal employment is common, income information systems are missing, and the cost and quality of income surveys are high (Coady et al., 2004; Zhang, 2017). Third, in addition to income level, group segmentation is highly intersectional or correlated with other social characteristics of individuals, including factors such as age, ethnicity, geography, and social capital. Objective criteria for either an interval of median income or an interval of absolute income level mostly ignore this correlation, and the choice of multiple indicators for mixed criteria only partially overcomes this deficiency (Ostrove et al., 2000; Ostrove and Cole, 2003). Combined with the reality of China, it is difficult to identify an objective criterion for defining middle-income groups that applies to both urban and rural areas and covers all regions due to the large differences in their economic development levels (Li and Xu, 2017).

While reflecting on the shortcomings of the objective criteria definition method, researchers seek other alternative or complementary methods. Drawing on the psychology of social class (PSG) findings, subjective methods of socioeconomic status evaluation have recently emerged in the field of social group-related research (Rubin et al., 2014; Yang et al., 2022).

The subjective evaluation method is a general evaluation of the individual’s relative position in the social hierarchy in terms of income level and social status with reference to others (Kraus et al., 2013). Compared with the objective criteria, the subjective evaluation method does not require an income household survey, does not rely on a specific definition interval, and is simple to implement, which can just compensate for the shortcomings of the objective criteria of middle-income groups (Adler and Stewart, 2014). The most representative measurement instruments for subjective assessment methods include the Single Item Measure of Perceived Social Status Category and the MacArthur Scale of Subjective Social Status. The single-item measure of the perceived social status category is usually administered by asking respondents about their relative economic and social status, for example, by dividing income into a few classes and asking them to rate where they fall in relation to other individuals in the country or community. With the MacArthur Subjective Social Class Scale measure, respondents are guided to evaluate their position on a 10-level “social ladder” by combining objective indicators such as their income level, education, and occupational prestige and comparing them to corresponding indicators for others in the country or community, with the higher the “social ladder,” the higher the individual’s social class.

The subjective assessment of socioeconomic status is simple and easy to administer, but its validity has been widely questioned. Typical questions include, first, whether individuals can identify their position on the “social ladder,” as measured by the MacArthur Subjective Social Class Scale, for example. If respondents are unable to identify their position on the “social ladder,” the dilemma faced by the subjective assessment method coincides with the shortcomings of objective criteria. Although subjective evaluation methods do not presuppose benchmarks to define different social classes or economic status, researchers can adopt contextualization strategies to guide respondents to reflect on and revise their internalized criteria. They can even manipulate the frame of reference so that respondents can make more precise assessments of their economic and social status (Kraus et al., 2009; Hamamura, 2012). Second, most respondents in the actual survey tend to evaluate themselves as a middle-income group, which in turn may lead to a large bias in the results (Ostrove and Long, 2007). To address such problems, Rubin et al. (2014) argued that dividing the middle-income group into several subcategories can basically overcome this possible shortcoming of the actual survey. Third, respondents’ emotions or emotional factors may affect the validity of subjective evaluation results. Kraus et al. (2009), Adler and Stewart (2014), and others found that experimentally manipulating respondents’ emotions did not change the subjective evaluations of their socioeconomic status.

In this research, we follow the hypothesis and research on on social class theory in Bourdieu (1984, 1985, 1987). That is, there are discrete social “class fragments” including lifestyles, social perceptions, political attitudes and political participation. Moreover, there are differences between different social classes and similarities within the same social class. Bourdieu (1984, 1985, 1987) initiated a series of studies on lifestyles, social perceptions, political attitudes and political participation of different social classes. For example, Brooks and Svallfors (2018), Manza and Crowley (2018) studied the voting behavior and preferences of different social groups, political attitudes toward government regulation, and public budgets. They found that people’s voting behavior and political attitudes are closely related to the position of individuals in the social class hierarchy. Easterly (2001), Brown (2004), and Esteban and Ray (2008, 2011) found that a stable and growing middle-income group is an important guarantee and driving force for social harmony and stability, economic growth, and poverty elimination. Boushey and Hersh (2012) summarize the paths through which middle-income groups contribute to economic growth and social stability: the promotion of human capital and the promotion of political participation. The paths to promote human capital and national education, a stable consumer of goods and services demand, a major source of future entrepreneurs, and a political and economic system that supports inclusion. Furthermore, studies on China also show that in the process of social transformation and change, social stratification is a classification of objective social location. Furthermore, it constitutes the basic dividing line of social relations and the basis of interests of different social groups, and to a considerable extent affects people’s residential differentiation, social interaction and social identity (Liu and Li, 2005).

However, many empirical studies have found differences between the subjective evaluation methods and objective criteria in different “scenarios.” For example, Cohen et al. (2008) found that not objective socioeconomic status but subjective evaluation (perception) of individual socioeconomic status has a substantial impact on their health status. Li et al. (2020) examined the rationality hypothesis of China’s status and showed that whether it is adults or adolescents, objective socioeconomic status and subjective socioeconomic status have completely opposite effects. Tan et al. (2020) showed a stronger relationship between social happiness and the subjective socioeconomic status than objective socioeconomic status. Buchel et al. (2021) found that objectively disadvantaged groups, rather than subjectively disadvantaged groups, have greater incentives to defend institutions.

The existing research does not judge the merits of the subjective evaluation method and the objective criteria. The objective criteria have their inherent defects. The subjective evaluation method can partially overcome the weaknesses of the objective standard and is relatively simple and easy to implement. Differences in factors such as diversity, environment, and culture, as well as differences in the selection of reference objects, may lead to hidden limitations and risks in the subjective evaluation results of socioeconomic status. Following Bourdieu (1984, 1985, 1987) and recognizing the differences between the subjective evaluation method and the objective criteria, we believe that the subjective evaluation method and the objective criteria are only appropriate for their own scenarios.

There is a vast literature on government trust. Mei and Tao (2018) and Zhao and Wang (2021) have provided comprehensive reviews and summaries, which will not be repeated here. There are several relevant studies on the differences in government trust among different income groups in China. Among them, Jing and Yang (2012) argued that in China, some new social classes are gradually formed, and the differences in social, economic, lifestyle and interest identities among the classes are becoming clearer, and the “social trust” shared by different social classes and social groups have their own characteristics. The study found that the peasant class has the highest trust in the government, followed by the working class, the old middle class, and the new middle class, while the owner class has the lowest trust in the government. Zou et al. (2020) studied the trust in local governments and found no difference between the subjective lower middle class and the subjective upper middle class, but the subjective middle class is significantly higher than the subjective lower middle class, showing an inverted “U” pattern.

In summary, the main research objectives of this paper include the following. First, we compare the differences between subjective evaluation methods and objective criteria based on measuring the size of middle-income groups. Second, based on the study of government trust in different income groups, we compare the differences in government trust in different income groups defined by subjective evaluation methods and objective criteria. Third, we test the validity of the results of the two methods in assessing government trust. Fourth, based on the possible shortcomings of subjective criteria, the stability and validity of the subjective evaluation results are examined, and the robustness of government trust in different income groups is tested.

## 3. Measuring the size of the middle-income group

### 3.1. Data sources

This paper conducts an empirical study using data from the China Family Panel Studies (CFPS) 2018, a national, comprehensive social tracking survey project that aims to reflect social, economic, demographic, educational, and health changes in China by tracking and collecting data at three levels: individual, household, and community.^2^

### 3.2. Comparison of subjective evaluation and objective standard measurement results

In this paper, we choose two objective criteria commonly used in the literature: the relative criterion defines the middle-income group in the range of 75–150% of median *per capita* household income (Long, 2012, 2015; Weng and Wang, 2019; Ruan et al., 2021), and the absolute criterion defines the middle-income group in the range of total annual household income of $69,000–236,000 (Li and Xu, 2017; Weng and Wang, 2019).^3^

The subjective evaluation of respondents’ income in the CFPS project was implemented through the following question in the questionnaire: “1 means very low, 5 means very high, how would you rate your income in your local area?” Respondents were defined as being in the low-, middle-, or high-income groups if they scored 1–2, 3, or 4–5, respectively.

Combining the measurements in Table 1, the following differences exist between the subjective evaluation of income and the objective criteria. The sizes of the low-income group and high-income group measured by relative criteria are larger than those measured by the subjective criteria. In addition, the size of the middle-income group is smaller than those measured by the subjective criteria. The size of the low-income group measured by the absolute standard is larger than that measured by the subjective evaluation method. In addition, the sizes of the middle-income group and the high-income group are smaller than that measured by the subjective criteria.

**Table 1:** Size measurement results of different income groups (unit: %).

Standard	Low-income group	Middle-income group	High-income group
Objective criteria (relative criteria)	39.43	26.81	33.76
Objective criteria (absolute criteria)	59.39	36.84	3.77
Subjective evaluation	30.00	46.94	23.07

At the same time, the study shows that there is only a weak positive correlation between the subjective evaluation of income and the objective criteria. In particular, the correlation coefficient between the subjective evaluation of income and the relative standard measure results was 0.0265, and the correlation coefficient with the absolute standard measure results was 0.0459. Still, both were significant at the 1% significance level. The findings are similar to those of Cohen et al. (2008), Tan et al. (2020), and others. For example, Cohen et al. (2008) found that subjective socioeconomic status showed a non-significant weak positive correlation (correlation coefficient of 0.06, *p* > 0.43) with objective criteria (measured by income) measures.

## 4. Study on the difference in government trust among different income groups

The survey of respondents’ trust in government in the CFPS project was implemented through the following question “Your overall evaluation of the work of government in your county or county city/district last year was: 1. had a great achievement, 2. had some achievement, 3. did not have much achievement, 4. had no achievement, and 5. was worse than before.” In this paper, we construct a ranking model for empirical study, and the explanatory variables are individuals’ overall evaluation of the work of local government (worse than before = 1, no achievement = 2, not much achievement = 3, some achievement = 4, and great achievement = 5).

The main explanatory variables are different income groups (low-income group, middle-income group, and high-income group) defined according to the subjective evaluation of individual income and objective criteria, respectively. The selection of control variables draws on studies such as Mei and Tao (2018) and Zhao and Wang (2021), combined with the survey content of the CFPS project, and includes: factors measuring individual characteristics or their own abilities, including gender, age, education level, and health status; factors measuring individual social relationships or social interaction abilities, including marital status, work status, whether they are Communist Party membership in organizations such as labor unions, religious belief groups, and individuals’ associations; factors measuring individuals’ access to information, including whether they use mobile devices to access the Internet, whether they use computers to access the Internet, and the amount of time they watch TV per day (logarithm); and two variables measuring individuals’ living environment, urban–rural and regional.

Combining the regression results in Table 2, the following conclusions were obtained. First, compared to individuals who subjectively rated themselves as the middle-income group, individuals who subjectively rated themselves as the low-income group had a significantly worse overall evaluation of local government, and individuals who subjectively rated themselves as the high-income group had a significantly better overall evaluation of local government.

**Table 2:** Regression results of trust in government for different income groups.

Explanatory variables	Subjective evaluation	Objective criteria	
	Government evaluation	Government evaluation (relative standard)	Government evaluation (absolute standard)
Low-income group	−0.245^***^ (−9.21)	0.0107 (0.37)	−0.0578^**^ (−2.25)
High-income group	0.169^***^ (5.68)	0.0391 (1.27)	0.0447 (0.72)
Gender (male)	−0.129^***^ (−5.42)	−0.136^***^ (−5.75)	−0.135^***^ (−5.70)
Age	−0.0223^***^ (−4.34)	−0.0244^***^ (−4.74)	−0.0244^***^ (−4.75)
Age squared	0.000383^***^ (7.46)	0.000413^***^ (8.06)	0.000413^***^ (8.07)
Education Level	0.0907^***^ (8.08)	0.0883^***^ (7.75)	0.0857^***^ (7.54)
Health Status	−0.111^***^ (−10.95)	−0.131^***^ (−13.00)	−0.130^***^ (−12.93)
Marital status (married)	−0.233^***^ (−4.87)	−0.212^***^ (−4.42)	−0.223^***^ (−4.66)
Marital status (divorced)	−0.134 (−1.47)	−0.149 (−1.63)	−0.148 (−1.63)
Marital status (widowed)	−0.0737 (−1.01)	−0.0895 (−1.23)	−0.0973 (−1.34)
Work status (active)	−0.0336 (−1.11)	−0.0156 (−0.52)	−0.0141 (−0.47)
Communist Party member	0.407^***^ (9.71)	0.424^***^ (10.11)	0.422^***^ (10.07)
Trade Union Members	0.199^***^ (4.63)	0.212^***^ (4.89)	0.208^***^ (4.81)
Members of religious faith groups	0.212^***^ (3.18)	0.215^***^ (3.22)	0.217^***^ (3.25)
Member of individuals’ association	0.123^**^ (2.27)	0.153^***^ (2.83)	0.154^***^ (2.86)
Mobile Internet	−0.141^***^ (−4.64)	−0.151^***^ (−4.96)	−0.154^***^ (−5.06)
Computer Internet access	0.135^***^ (3.86)	0.142^***^ (4.05)	0.139^***^ (3.94)
TV viewing time (logarithmic)	0.0426^***^ (3.71)	0.0420^***^ (3.66)	0.0414^***^ (3.62)
Region (Central)	−0.0348 (−1.15)	−0.0342 (−1.12)	−0.0312 (−1.02)
Region (West)	0.0221 (0.74)	0.0310 (1.03)	0.0369 (1.23)
Region (Northeast)	−0.416^***^ (−11.29)	−0.420^***^ (−11.38)	−0.410^***^ (−11.00)
Urban and rural (rural)	0.0762^***^ (3.08)	0.0921^***^ (3.64)	0.0952^***^ (3.82)
LR test value	1314.75 (*p* = 0.0000)	1145.09 (*p* = 0.0000)	1149.62 (*p* = 0.0000)
Pseudo *R*^2^	0.0450	0.0391	0.0392
Number of samples	27,003	27,003	27,003

^*^, ^**^, and ^***^ indicate significant at 10, 5, and 1% significance levels, respectively, and *t*-statistic values are in parentheses. Gender is taken as the reference for females; education level (1 = illiterate/semi-literate, 2 = elementary, 3 = junior high, 4 = high school/junior college/technical school/vocational high, 5 = junior college, 6 = undergraduate, 7 = master, 8 = doctoral) is regressed as a continuous variable and the findings are similar to the regression results as a categorical variable; health status (1 = very healthy, 2 = very healthy, 3 = healthy, 4 = fair and 5 = unhealthy), which are also regressed as continuous variables in this paper; marital status is referenced as unmarried; job status is referenced as jobless, unemployed or withdrawn from the labor market; regional is referenced as eastern region, and urban and rural is referenced as urban.

Second, there is no significant difference in the overall evaluation of local governments by different income groups defined according to relative criteria. Compared to the middle-income group, the overall evaluation of local governments by the low-income group defined by absolute criteria is significantly worse. The overall evaluation of local governments by the high-income group is not significantly different from that of the middle-income group.

Third, the effect of individual other explanatory variables on the overall evaluation of local government is generally stable. Compared with females, males have a worse overall evaluation of local government. The relationship between an individual’s age and their overall evaluation of local government is “U” shaped, i.e., as an individual grows older, their overall evaluation of local government gradually becomes worse, and then gradually becomes better after reaching a certain age. Individuals with higher education levels have a significantly better overall evaluation of local government. Individuals with poorer health status also rated local governments worse overall. Married individuals rated local government worse overall compared to unmarried individuals. Individuals who are members of organizations such as the Communist Party, trade unions, religious faith associations, and individuals’ associations all have a better overall evaluation of local government. Individuals rated local governments poorly overall if they used mobile devices to access information online. They rated local governments better overall if they used computers to access information online or watched television for a more extended time each day. Compared to the Eastern region, individuals in the Northeastern region rated the local government less favorably overall. In contrast, individuals in the rural areas rated the local government more favorably overall compared to the urban areas.

In summary, if the overall evaluation of local government is used as a measure of government trust, there are significant differences in government trust among different income groups defined based on subjective evaluation methods. However, the differences in government trust among different income groups defined based on objective criteria are generally insignificant.

## 5. Robustness test

### 5.1. Robustness of subjective evaluation results

Although the subjective evaluation method of socioeconomic status is simple and easy to implement, its validity and stability need to be double-checked. For example, respondents’ emotions or emotional factors may affect the subjective evaluation results. In this paper, it is also necessary to test the stability and reliability of the subjective evaluation results.

The CFPS project also included a survey on respondents’ self-assessment of their social status “On a scale of 1 for very low and 5 for very high, how would you rate your social status in your local area?” Logically, if a respondent scores high on the subjective assessment of income, he or she should also score high on the subjective assessment of social status. We matched respondents’ income subjective evaluation scores with their social status subjective evaluation scores (Table 3), and we can see that the percentage of exact matches between the two is quite high. For example, 8,355 of the 12,600 samples (66.31%) with a subjective evaluation score of 3 on social status also had a subjective evaluation score of 3 on income. Also, the study showed that the reliability test value (Cronbach’s alpha) of the two was 0.6812, and the correlation coefficient was 0.5171 (significant at 1% significance level). It is generally believed that if Cronbach’s alpha is higher than 0.7, it indicates that the questionnaire survey content has a fairly high consistency and reliability, and if the value of this coefficient is lower than 0.6, it indicates that the consistency and reliability of the questionnaire survey are insufficient. The Cronbach’s alpha obtained in this paper is slightly lower than 0.7 but much higher than 0.6, which verified the stability and validity of the subjective evaluation results.

**Table 3:** Matching of subjective evaluation of income with subjective evaluation of social status.

Subjective evaluation of income	Subjective evaluation of social status			
	1–2 points	3 points	4–5 points	Total
1–2 points	4,345	2,699	1,197	8,241
3 points	1808	8,355	2,768	12,931
4–5 points	408	1,546	4,399	6,353
Total	6,561	12,600	8,364	27,525

### 5.2. Robustness of differences in government trust across income groups

As argued by Mei and Tao (2018) and Zhao and Wang (2021), government trust can be measured from multiple perspectives, which include the level of trust in government officials. The CFPS project’s trust in local government officials was implemented through the following question “If a score of 0 means very little trust and a score of 10 means a lot of trust, how much do you/you trust local government officials?” Logically, if respondents have a better overall rating of local government, they should have a higher level of trust in local government officials (higher score), and the study shows that the reliability test (Cronbach’s alpha) is 0.4025 and the correlation coefficient is 0.3976 (significant at 1% significance level), indicating that the findings are trustworthy.

We construct an empirical study using respondents’ trust in local government officials as the explanatory variable and construct a ranking model to test the robustness of government trust and influencing factors across income groups. At the same time, there may be endogeneity problems caused by omitted variables and other factors influencing government trust in the empirical study. We exclude the sample with only one family member and construct a household fixed effects model to conduct an empirical study to partially overcome the possible endogeneity problem and test the robustness of government trust and influencing factors across income groups.

Comparing the results in Tables 2, 4, 5, the robustness of trust in government and other influencing factors across income groups was verified. Among them, individuals with subjective ratings of low-income groups have significantly lower trust in local government officials. Individuals with subjective ratings of high-income groups have significantly higher trust in local government officials relative to individuals with subjective ratings of middle-income groups. The household fixed-effects model with the evaluation of local government in general as the explanatory variable yields similar findings. Similar to the regression results in Table 2, there is no significant difference in trust in local government officials among different income groups defined by absolute criteria. However, there is a significant difference in trust in local government officials among different income groups defined by the relative criteria, which differs from the regression results in Table 2.

**Table 4:** Robustness tests of trust in government for different income groups.

Explanatory variables	Subjective evaluation	Objective criteria	
	Officer trust	Officer trust (relative standard)	Officer trust (absolute standard)
Low-income group	−0.436^***^ (−17.10)	0.0494^*^ (1.78)	0.00867 (0.36)
High-income group	0.299^***^ (10.70)	−0.0503^*^ (−1.72)	−0.0116 (−0.21)
Gender (male)	−0.212^***^ (−9.41)	−0.229^***^ (−10.18)	−0.228^***^ (−10.10)
Age	−0.0244^***^ (−5.04)	−0.0266^***^ (−5.49)	−0.0275^***^ (−5.68)
Age squared	0.000430^***^ (8.92)	0.000474^***^ (9.83)	0.000480^***^ (9.97)
Education level	0.0162 (1.54)	0.0225^**^ (2.10)	0.0168 (1.57)
Health status	−0.103^***^ (−10.69)	−0.139^***^ (−14.55)	−0.138^***^ (−14.44)
Marital status (married)	−0.328^***^ (−7.28)	−0.303^***^ (−6.71)	−0.295^***^ (−6.53)
Marital status (divorced)	−0.262^***^ (−3.05)	−0.291^***^ (−3.39)	−0.288^***^ (−3.35)
Marital status (widowed)	−0.215^***^ (−3.16)	−0.253^***^ (−3.73)	−0.246^***^ (−3.63)
Work status (active)	0.0848^***^ (2.98)	0.112^***^ (3.94)	0.114^***^ (4.00)
Communist party member	0.341^***^ (8.79)	0.380^***^ (9.79)	0.377^***^ (9.70)
Trade union members	0.0875^**^ (2.19)	0.130^***^ (3.24)	0.117^***^ (2.92)
Members of religious faith groups	0.101 (1.62)	0.108^*^ (1.74)	0.108^*^ (1.74)
Member of individuals’ association	0.0580 (1.12)	0.115^**^ (2.23)	0.115^**^ (2.23)
Mobile internet	−0.237^***^ (−8.18)	−0.245^***^ (−8.42)	−0.252^***^ (−8.69)
Computer internet access	0.121^***^ (3.66)	0.150^***^ (4.51)	0.143^***^ (4.30)
TV viewing time (logarithmic)	0.0585^***^ (5.39)	0.0604^***^ (5.55)	0.0580^***^ (5.34)
Region (Central)	−0.00318 (−0.11)	−0.0241 (−0.83)	−0.0133 (−0.46)
Region (West)	−0.00465 (−0.17)	−0.0149 (−0.52)	−0.000700 (−0.02)
Region (Northeast)	−0.334^***^ (−9.41)	−0.350^***^ (−9.85)	−0.346^***^ (−9.67)
Urban and rural (rural)	0.239^***^ (10.18)	0.240^***^ (9.95)	0.256^***^ (10.78)
LR test value	2175.70 (*p* = 0.0000)	1599.11 (*p* = 0.0000)	1587.82 (*p* = 0.0000)
Pseudo *R*^2^	0.0777	0.0585	0.0580
Number of samples	27,373	27,373	27,373

^*^, ^**^, and ^***^ denote significance at the 10, 5, and 1% significance levels, respectively, with *t*-statistics in parentheses.

**Table 5:** Robustness tests of trust in government using household fixed effects model.

Explanatory variables	Government evaluation	Officer trust
Low-income group	−0.107^***^ (−6.01)	−0.481^***^ (−10.06)
High-income group	0.0524^***^ (2.74)	0.338^***^ (6.64)
Gender (male)	−0.0459^***^ (−3.58)	−0.250^***^ (−7.42)
Age	−0.0101^***^ (−2.61)	−0.0208^**^ (−2.01)
Age squared	0.000165^***^ (4.22)	0.000518^***^ (4.94)
Education level	0.0504^***^ (6.05)	0.0890^***^ (3.97)
Health status	−0.0408^***^ (−5.80)	−0.108^***^ (−5.68)
Marital status (married)	−0.0834^**^ (−2.22)	−0.374^***^ (−3.83)
Marital status (divorced)	−0.101 (−1.25)	−0.239 (−1.20)
Marital status (widowed)	−0.0489 (−0.85)	−0.333^**^ (−2.19)
Work status (active)	−0.0702^***^ (−3.29)	−0.0776 (−1.36)
Communist party member	0.120^***^ (4.68)	0.268^***^ (3.92)
Trade union members	0.111^***^ (3.82)	0.0686 (0.92)
Members of religious faith groups	0.0571 (1.09)	0.0142 (0.11)
Member of individuals’ association	0.0456 (1.19)	0.0396 (0.39)
Mobile internet	−0.0604^***^ (−2.79)	−0.187^***^ (−3.27)
Computer internet access	0.0427^*^ (1.81)	0.170^***^ (2.72)
TV viewing time (logarithmic)	0.0174^**^ (2.11)	0.0516^**^ (2.33)
Cons	3.605^***^ (41.69)	5.358^***^ (22.89)
F test value	19.34 (*p* = 0.0000)	37.03 (*p* = 0.0000)
*R* ^2^	0.0433	0.0768
Number of samples	22,410	22,410

^*^, ^**^, and ^***^ denote significance at the 10, 5, and 1% significance levels, respectively, with *t*-statistics in parentheses. The household fixed effects model in which different members of the same household are located in exactly the same, urban–rural and regional areas are automatically excluded from the model regression.

## 6. Conclusion

This paper measures and compares the size of middle-income groups in China based on the subjective evaluation of income and the objective criteria using survey data from the China Household Tracking Survey Project 2018. Furthermore, the differences in government trust of different income groups defined by subjective evaluation and the objective criteria are examined. Synthesizing the research in this paper, we have the following major findings.

First, there are considerable differences between the results of the subjective evaluation of income and objective criteria. There is only a weak positive correlation between the two. In practice, although the choice of the objective criteria for defining middle-income groups is controversial or even flawed, if the established criteria are determined, the results mainly depend on the location of individual income (absolute or relative income level) in the overall income distribution. As argued in the binary structure theory of social class constructed by Bourdieu (1984, 1985, 1987), in addition to the objective criteria, the subjective evaluation of income is also related to other objective economic resources under the control of the individual and their self-perceived position in the social hierarchy (Kraus et al., 2012; Manstead, 2018).

The findings of this paper are similar to those of Fan and Chen (2015) but differ from those of Lu and Zhang (2006). Fan and Chen (2015) used data from a large-scale microsurvey in China from 2003 to 2012 to classify class status identity bias into “consistent,” “upward biased” and “downward biased.” They found that the three corresponding proportions were 29.14, 39.74 and 31.11%, respectively, And the class identity bias was more severe. Using research data from rural areas in Zhejiang Province, China, Lu and Zhang (2006) found that the correlation coefficients between the subjective identity of household economic status and social status and the stratification status of total annual household income were 0.511 and 0.418, respectively. This could be due to the relatively small sample size (350) used, and the survey was limited to economically developed. This may be due to the small sample size (350). Furthermore, the survey was limited to rural areas in economically developed regions, resulting in a high degree of consistency between subjective economic and social status identity and objective economic status stratification.

Class status identification bias is more prevalent in China than in developed countries. For example, Sosnaud et al. (2013) found that more than half of the U.S. population can identify their class social status more accurately. Hence, the percentage of Chinese class status identification and objective class congruence is lower than that of the U.S. (Fan and Chen, 2015). The reasons for the higher class status identity bias in China may include the following. First, China’s economy and society are in a stage of rapid transition and change. Compared to the pre-reform and opening-up period and developed countries, the differences in income level, education level, and occupational prestige of individuals or families have been expanding, and the mobility of social classes has been increasing, which inevitably brings impacts on the consistency of class status perceptions. Second, Chinese traditional culture may also be the reason for higher class status identity bias. For example, the traditional Chinese culture is based on the virtue of understatement and modesty, and individuals with higher education, income level or professional prestige are more likely to underestimate their subjective socioeconomic status. Third, under similar objective conditions, individuals choose different reference objects and perceive the objective conditions differently. For example, two individuals living in a closed environment and an open and mobile environment with similar objective conditions may differ in the reference objects they choose, inevitably leading to differences in the perception of subjective class status. The large differences in economic and social development levels between urban and rural areas and between regions in China may also be the reason for the large bias in the perception of social class in China.

Second, relative to the objective criteria, there is a more significant association between the subjective evaluation of income and trust in government. If the overall evaluation of local government or trust in local government officials is used as a measure of trust in government, there are significant differences in trust in government among different income groups defined based on the subjective evaluation methods. Individuals in the higher income group have a significantly better overall evaluation of local government and a considerably higher trust in local government officials. However, the differences in trust in government among different income groups defined based on the objective criteria are insignificant overall. The above findings are similar to those of Cohen et al. (2008), Li et al. (2020), Tan et al. (2020), and Buchel et al. (2021) in that it is not objective socioeconomic status but rather the subjective evaluation of individual socioeconomic status that makes a substantial difference in trust in government.

There is a paucity of research on the differences in trust in government across income groups as defined by subjective assessments of socioeconomic status and objective standard measures in China. However, related studies on other topics in China have yielded similar findings to this paper. For example, Cai and Wang (2018) found that the relatively lower objective social status but higher subjective well-being of rural residents than urban residents in China is an important reason for the relatively higher subjective social status of rural residents. Theoretically, Akerlof and Kranton (2000) and Costa-Font and Cowell (2015) introduced identity into the neoclassical analytical framework. Social identity has been considered a key determinant of endogenous economic behavior and preferences, with very important implications for explaining both redistributive preferences and pro-social behavior. In particular, under the constraints of information scarcity and limited rationality, individual behaviors and preferences are more likely to be influenced by subjective perceptions of socioeconomic status than by objective socioeconomic status.

In practice, the objective criteria for defining middle-income groups are still widely debated in both developed and developing countries, and the choice of objective criteria faces even more difficulties based on the realities of China. The different objectives criteria may lead to the dilemma of ambiguity in the objectives of policies to expand the middle-income group. Moreover, the perceived socioeconomic status of individuals may be a key factor guiding their behavior. When the results of objective criteria (reality) are not consistent with the subjective evaluation of individuals’ socioeconomic status (perception), the “label” of the group defined by objective criteria may lose its meaning. That is, the effectiveness of policies targeting the group defined by objective criteria may be greatly reduced. Of course, the subjective evaluation method can overcome the defects of objective criteria and is relatively simple and easy to implement, but it also has inherent defects or shortcomings. This paper argues that the subjective evaluation methods can be considered as a supplement or alternative to the objective criteria for defining middle-income groups in China. This is true especially when the objective criteria are difficult to determine or are highly controversial. The research in this paper provides a major policy insight. That is, to improve people’s trust in the government, there may be a dilemma in targeting different income groups defined by the objective criteria.

## Data availability statement

The original contributions presented in the study are included in the article/supplementary material, further inquiries can be directed to the corresponding author.

## Author contributions

All authors listed have made a substantial, direct, and intellectual contribution to the work and approved it for publication.

## Funding

This paper is supported by the China National Social Science Foundation’s project “Research on the size measurement and influence mechanism of middle-income group” (Project #: 17BJY042).

## Conflict of interest

The authors declare that the research was conducted in the absence of any commercial or financial relationships that could be construed as a potential conflict of interest.

## Publisher’s note

All claims expressed in this article are solely those of the authors and do not necessarily represent those of their affiliated organizations, or those of the publisher, the editors and the reviewers. Any product that may be evaluated in this article, or claim that may be made by its manufacturer, is not guaranteed or endorsed by the publisher.

## Footnotes

^1^Manipulating subjects’ subjective class, instead of changing their true social class status, researchers can simply apply different manipulations to subjects in different experimental groups to make some subjects temporarily feel their social class is higher and others temporarily feel their social class is lower, so that the effect of social class on individual psychology and behavior can be analyzed at the causal level (Yang et al., 2022).

^2^http://www.isss.pku.edu.cn/cfps/index.htm for data details and descriptions.

^3^The absolute criteria tried to be used in this paper also include: total annual household income of 100,000-500,000 yuan (National Bureau of Statistics standard); annual per capita household income of 24,000-240,000 yuan (World Bank daily per capita income of $10-100 standard); and annual per capita household income of 22,000-65,000 yuan (Li and Wang, 2014; National Development and Reform Commission Social Development Institute group, 2012). However, the size of high-income groups measured according to the above criteria are very low and do not have research value.
